# IMPACT web portal: oncology database integrating molecular profiles with actionable therapeutics

**DOI:** 10.1186/s12920-018-0350-1

**Published:** 2018-04-20

**Authors:** Jennifer D. Hintzsche, Minjae Yoo, Jihye Kim, Carol M. Amato, William A. Robinson, Aik Choon Tan

**Affiliations:** 1 Division of Medical Oncology, Department of Medicine, Translational Bioinformatics and Cancer Systems Biology Laboratory, University of Colorado Anschutz Medical Campus, Aurora, CO USA; 2 Division of Medical Oncology, Department of Medicine, Robinson Melanoma Laboratory, University of Colorado Anschutz Medical Campus, Aurora, CO USA

**Keywords:** Next generation sequencing, Oncology, Therapeutics, Drug repurposing

## Abstract

**Background:**

With the advancement of next generation sequencing technology, researchers are now able to identify important variants and structural changes in DNA and RNA in cancer patient samples. With this information, we can now correlate specific variants and/or structural changes with actionable therapeutics known to inhibit these variants. We introduce the creation of the IMPACT Web Portal, a new online resource that connects molecular profiles of tumors to approved drugs, investigational therapeutics and pharmacogenetics associated drugs.

**Results:**

IMPACT Web Portal contains a total of 776 drugs connected to 1326 target genes and 435 target variants, fusion, and copy number alterations. The online IMPACT Web Portal allows users to search for various genetic alterations and connects them to three levels of actionable therapeutics. The results are categorized into 3 levels: Level 1 contains approved drugs separated into two groups; Level 1A contains approved drugs with variant specific information while Level 1B contains approved drugs with gene level information. Level 2 contains drugs currently in oncology clinical trials. Level 3 provides pharmacogenetic associations between approved drugs and genes.

**Conclusion:**

IMPACT Web Portal allows for sequencing data to be linked to actionable therapeutics for translational and drug repurposing research. The IMPACT Web Portal online resource allows users to query genes and variants to approved and investigational drugs. We envision that this resource will be a valuable database for personalized medicine and drug repurposing. IMPACT Web Portal is freely available for non-commercial use at http://tanlab.ucdenver.edu/IMPACT.

## Background

Next generation sequencing of cancer genomes has revolutionized the field of precision oncology in recent years. Using this technology, it is now possible to classify cancer subtypes based on the similarity of their molecular profiles. With biomarkers-driven clinical trials such as NCI-MATCH [1], it is now possible to treat specific genomic profiles of tumors regardless of their cancer type. This revolution of genomic-based therapeutics has advanced the march towards legitimate precision oncology. However, there is a need for researchers to be able to have a resource to query molecular profiles and connect those profiles to approved or investigational therapeutics.

Here we present IMPACT Web Portal, a database linking the molecular profiles of tumors to clinical and pre-clinical oncology actionable therapeutics. We previously published a whole exome sequencing (WES) analysis pipeline, IMPACT (Integrating Molecular Profiles with ACtionable Therapeutics) that matches molecular profiles with actionable therapeutics [2]. However, this tool is currently only available for WES data and requires command line-level programming skills. To facilitate the translational ability of this method, we developed a web-based database that requires no programming skills and is applicable to any type of sequencing data source.

Several databases have been developed to provide drug-gene interactions [3–6], some of the databases are focusing in cancer with drug-target gene variants information [7–10]. The IMPACT Web Portal differs from existing resources in the following aspects: (i) IMPACT web portal includes actionable therapeutics integrated from ten different data sources and includes all approved oncology drugs, a variety of current investigational drugs in cancer clinical trials, and pharmacogenetics databases. (ii) IMPACT Web Portal allows for users to input molecular profiles of individual tumors. The search can include genes, variants, fusions, and copy number changes that will each link to all known actionable therapeutics in the database. (iii) IMPACT Web Portal uses a drug-based database to rank potentially therapeutic compounds into 3 levels: Level 1 contains all approved drugs with variant-level (Level 1A) and gene-level (Level 1B) evidence. Level 2 contains drugs currently in cancer clinical trials. Level 3 uses pharmacogenetics to link altered gene targets to potentially actionable therapeutics. (iv) A hypergeometric test is used to calculate a *p*-value in order to rank each drug by its specificity to the molecular profile. (v) IMPACT Web Portal links information to other resources for continued investigation of drug-gene interactions. Each drug name is a link, taking users to a drug-oriented results page listing other gene targets of the drug, and when available the structure and PubChem identification number of the drug. Each gene also links to the external NCBI gene database. (iv) IMPACT Web Portal has the largest collection of genes, variants, fusions, and copy number changes, linked to the largest number of actionable therapeutics when compared to other oncology databases. Here, we describe the IMPACT Web Portal, an online, user-friendly, database that connects a tumor’s molecular profile to actionable therapeutics integrated from ten of the most well curated data sources. We also provide an example illustrating the utility of the IMPACT Web Portal.

## Construction and content

### IMPACT database construction

**Fig. 1:**
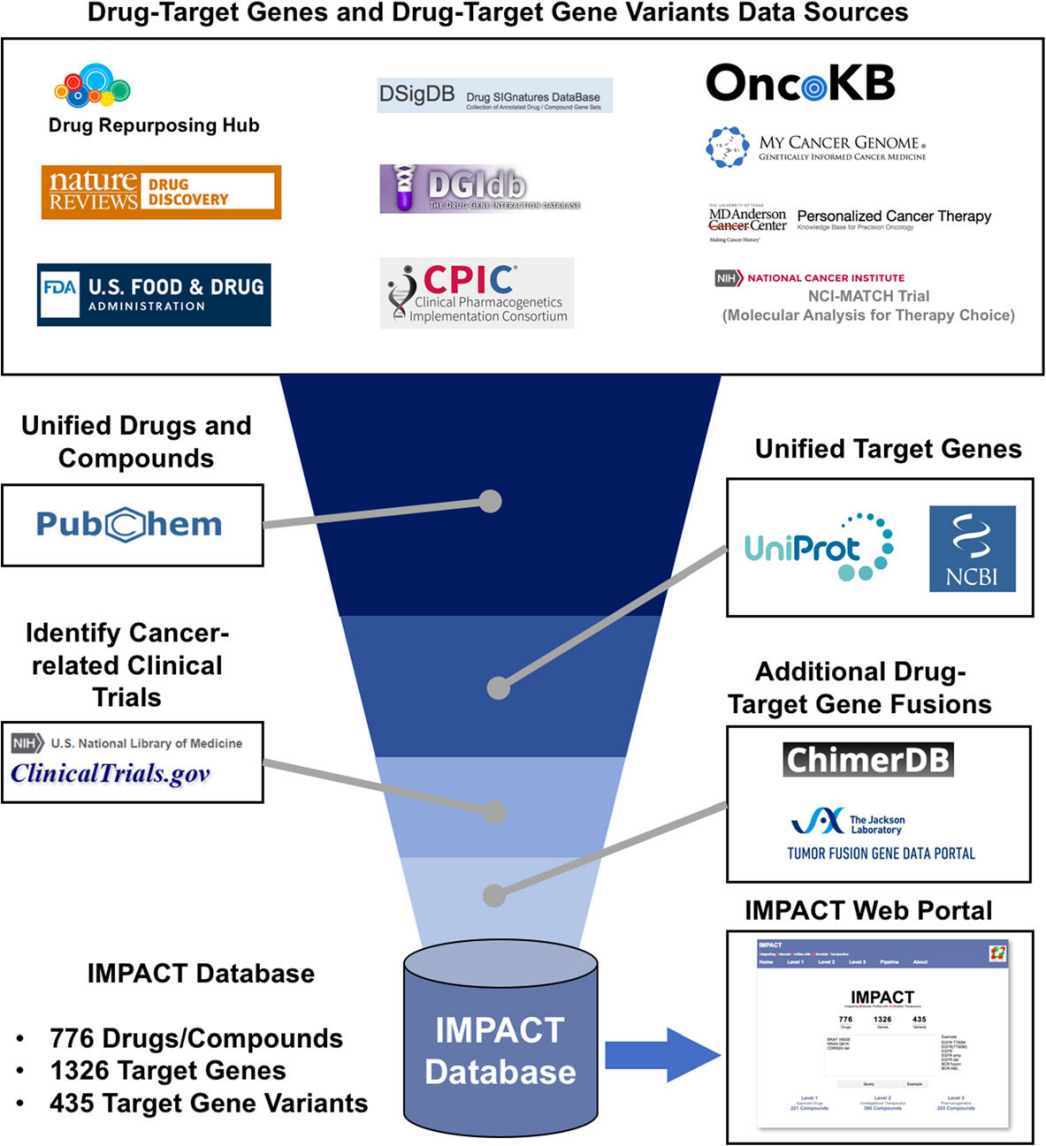
Development workflow of the IMPACT Database and Web Portal

Figure 1 illustrates the development workflow of the IMPACT database. We extracted drug-target genes and variants from ten data sources: the Drug Repurposing Hub [3], Food and Drug Administration (FDA) website (FDA.gov) [11], A Comprehensive Map of Molecular Drug Targets of FDA-approved drugs published in Nature Reviews Drug Discovery [4], DSigDB [6], DGIdb [5], OncoKB [7], My Cancer Genome [8], the MD Anderson Precision Cancer Therapy database [10], drug-gene relationships in the National Cancer Institute (NCI) MATCH clinical trials [1, 9], and Clinical Implementation of Pharmacogenetics Consortium (CPIC) [12]. Table 1 provides the descriptions of these data sources. We retrieved all the synonyms and International Chemical Identifier (InChI) or InChIKey of the compiled compounds list from PubChem [13]. We used InChI and InChIKey to identify and unify compounds in the list. To unify target genes and proteins, we used UniProt [14] to convert protein names to NCBI Entrez Gene Symbols [15]. For drugs that target gene fusions, we extracted additional known gene fusions from ChimerDB [16] and the Tumor Fusion Gene Data Portal [17]. We then queried the drugs list against ClinicalTrials.gov to retrieve all drugs tested in cancer clinical trials. Through these steps, we collected 776 drugs, 1326 target genes and 435 target gene variants. We developed the IMPACT database using MySQL version 14.14 (Distribute 5.7.11) on the OSX 10.11 (x86_64) platform. We used Python Version 2.7.11 to write scripts to perform data wrangling.

**Table 1:** Data Sources used in IMPACT Web Portal Database Construction

Data Source	Description	Reference
OncoKB	Precision oncology knowledge base of 100 drugs and their 476 genes and 3753 variants.	[7]
CPIC	Pharmacogenetic knowledge of drug-gene interactions of 203 drugs.	[12]
MD Anderson Precision Medicine	Contains 416 approved and investigational oncological drugs and their target genes.	[10]
My Cancer Genome	Contains 237 approved and investigational oncological drugs and their target genes.	[8]
Nature Reviews Drug Discovery: A compre-hensive map of molecular drug targets	A comprehensive mapping of 1578 US FDA-approved drugs and their 667 human targets.	[4]
Food and Drug Administration (FDA.gov)	Contains drug labelling of genes and variants for approved drugs.	[11]
The Drug Repurposing Hub	Extensive annotations of drugs and the genes they target for drug repurposing research. The current version contains 5628 compounds targeting 2172 proteins.	[3]
DSigDB	Approved and investigational therapeutics of drug gene signatures collected from PubChem/ChEMBL and kinase inhibition experiments.	[6]
DGIdb	Drug-gene interactions database collected from ten databases and 41 gene categories.	[5]
NCI-MATCH Trial	Drugs and associated target genes used to recruit various cancer patients (ClinicalTrials.gov NCT02465060).	[1, 9]
Tumor Gene Fusion Data Portal	Data base contains 8695 gene fusions detected from the Cancer Genome Atlas RNA-sequencing data.	[17]
ChimerDB	Comprehensive database of 1066 gene fusions encompassing analysis of RNA-sequencing data, PubMed Abstract text mining and manual curations.	[16]

### IMPACT database content

**Fig. 2:**
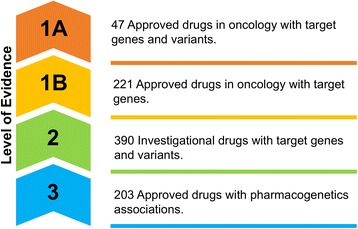
Three levels of evidence of drug-target genes in the IMPACT Web Portal. Level 1 contains Approved drugs; Level 2 contains Investigational therapeutics and Level 3 comprises pharmacogenetics information

We classified the drugs and compounds collected in the IMPACT database into three levels, based on the level of evidence of drug-target genes (Fig. 2).

#### Level 1 approved drugs

Level 1 contains 221 approved oncology drugs as of Sept 20, 2017. Level 1 is further divided into two sub levels: Level 1A comprises approved drugs with approved drug-target gene variants (including mutations, amplification, deletion and fusions); Level 1B consists of approved drugs with known target genes. Level 1A is composed of 47 approved drugs targeting 47 genes and 265 gene variants, fusions or copy number changes. Level 1B contains 221 oncological approved drugs that target 1170 genes.

#### Level 2 investigational therapeutics

Level 2 consists of 390 drugs currently being tested in cancer clinical trials (as of Sept 20, 2017). This set of investigational therapeutics target 370 genes.

#### Level 3 pharmacogenetics

Level 3 contains 203 approved drugs and their interactions with 110 genes obtained from the Clinical Pharmacogenetics Implementation Consortium (CPIC).

### IMPACT web portal

**Fig. 3:**
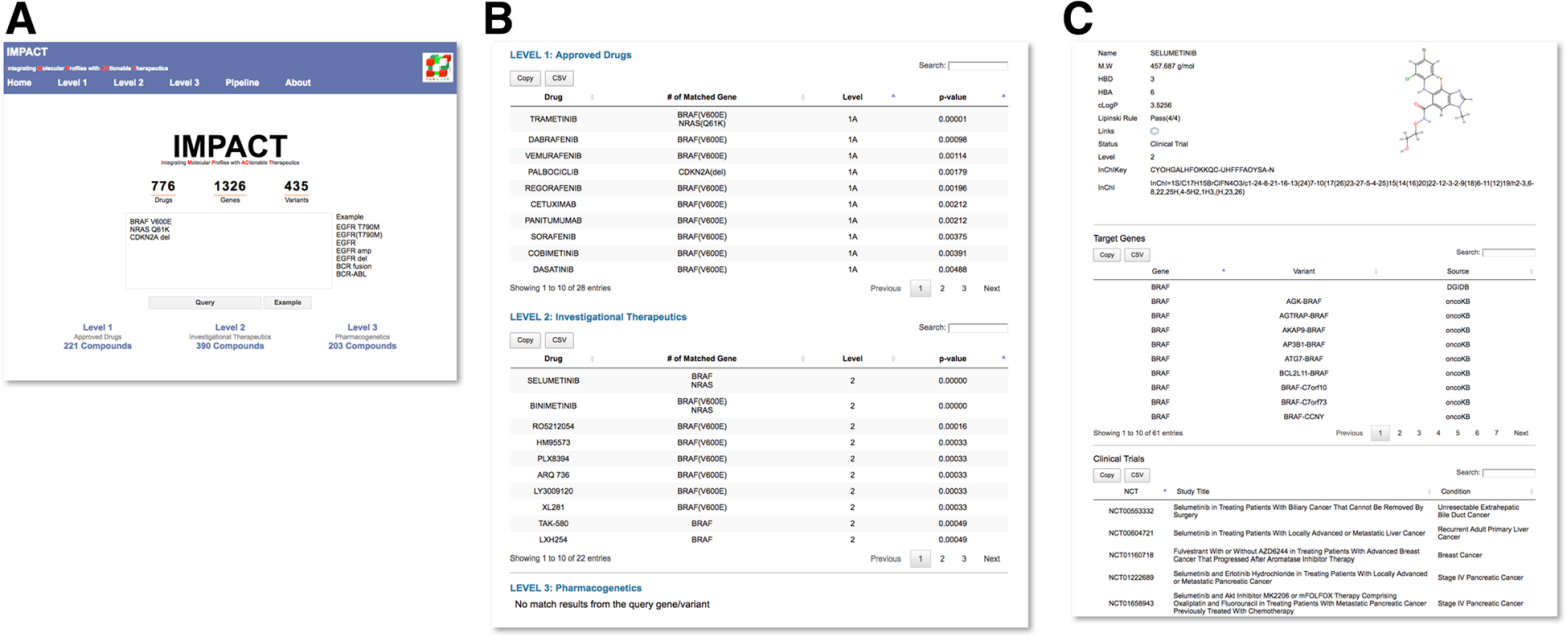
IMPACT Web Portal. **a** Query page. User could query single gene and variant, or query a list of genes and variants in the query box. The syntax for query genes are listed next to the query box. **b** Results page. Query genes and variants are matched with the IMPACT database, and returned as results in three levels: Level 1: Approved drugs; Level 2: Investigational therapeutics and Level 3: Pharmacogenetics drugs. The list is sorted by hypergeometric *p*-value. **c** Drug/compound page. By clicking the drug/compound in the results page, it will link to the drug/compound page. For each drug/compound page, the top part of the page provides molecular information about the drug/compound, with external link out to PubChem. The middle part of the page provides the list of target genes and variants, and the database sources where these drug-target genes and variants were collected in the IMPACT database. The bottom part of the page provides the list of clinical trials of the drugs/compounds, with link out to ClinicalTrials.gov

We developed a web portal and user interface to query IMPACT database using JavaScript and jQuery (Fig. 3a). The IMPACT Web Portal allows users to query, search, view and download data. In the query box, users are required to enter at least one gene (official gene symbol) followed by an optional alteration which can include a variant, copy number change, fusion, or fusion partner. For example, a user may enter any or all of the following on separate lines: BRAF, BRAF(V600E), BRAF V600E, BRAF(AMP), BRAF(fusion). Each entry will be queried in the database and mapped to potential actionable therapeutics in the IMPACT database. A hypergeometric test is conducted to compute a *p*-value for each mapped drug-target genes and drug-target gene variants.

Results from the query were returned in the IMPACT results page (Fig. 3b). The results page is divided into three levels based on the level of evidence. Within each level, each drug is listed followed by the mapped actionable genes and variants. Mapped drugs within each level were sorted by p-value (Fig. 3b). We developed a compound page to provide additional information for the drug (Fig. 3c). For the top part of the compound page, we used RDKit to generate the molecular descriptors for the compound. We used Marvin Sketch to draw the molecular structure. External links to PubChem is also provided in the compound page. The middle part of the compound page provides the target genes and variants of the drug, as well as the data source of the drug-target gene interactions. The bottom part of the compound page provides the list of Clinical Trials investigated by the compound.

### Data availability

IMPACT Web Portal is freely available for non-commercial research only use at http://tanlab.ucdenver.edu/IMPACT. IMPACT Web Portal data is available to download as tab-delimited plain text (.txt) files.

## Utility and discussion

To illustrate the utility of the IMPACT Web Portal, we performed a query from the analysis of whole-exome sequencing (WES) data. Previously, we published our IMPACT WES analysis pipeline using a melanoma case study where the patient has acquired resistant to dabrafenib and trametinib combination treatment [2]. Initially the patient tumor harbored the BRAF V600E mutation (Fig. 3) and was treated with vemurafenib (BRAF inhibitor). Following vemurafenib, the patient acquired resistant to this treatment with the development of the NRAS Q61K mutation. The patient was then treated with the combination of dabrafenib and trametinib. The patient acquired resistant to this combination therapy after 2 years, and the resistant tumor of this stage has acquired an additional CDKN2A deletion. To identify potential treatment for the patient at the resistant to dabrafenib and trametinib, we queried the three driver- and acquired- mutations (BRAF V600E, NRAS Q61K and CDKN2A deletion) to IMPACT Web portal. As illustrated in Fig. 3b, this query returns ten drugs in Level 1A. Among these ten approved drugs, palbociclib is the only drug that target CDKN2A deletion, whereas the other nine drugs are targeting BRAF V600E or NRAS Q61K. Since the patient already resistant to drugs that target BRAF and NRAS, the potential treatment for this patient maybe palbociclib. Interestingly, recent preclinical study has demonstrated that the combination of MEK inhibitor with palbociclib is an effective treatment in NRAS-mutant melanoma [18]. This finding warrants further preclinical and clinical evaluation to treat melanoma patients that acquired resistant to dabrafenib and trametinib.

## Conclusions

In conclusion, we developed IMPACT Web Portal, a novel online database for connecting molecular profiles to actionable therapeutics. The IMPACT Web Portal online resource allows users to search and connect 1326 target genes and 435 target gene variants against 776 approved and investigational cancer drugs. By utilizing three distinct levels of actionable therapeutics, users are able to find drugs already approved (Level 1), currently being tested in clinical trials (Level 2), and with pharmacogenetic evidence (Level 3). We believe that IMPACT Web Portal represents a significant improvement in the ability to connect molecular profiles with actionable therapeutics by using up to date resources. The user-friendly IMPACT Web Portal allows users to search for molecular profiles of individual tumors from any sequencing data and match them to actionable therapeutics for translational or drug repurposing oncology studies.

## Availability and requirements

The IMPACT Web Portal is freely available to all users at http://tanlab.ucdenver.edu/IMPACT. This web portal is accessible by web browser.

## Abbreviations

CPIC: Clinical Implementation of Pharmacogenetics Consortium
FDA: Food and Drug Administration
IMPACT: Integrating Molecular Profiles with Actionable Therapeutics
InChI: International Chemical Identifier
NCI: National Cancer Institute
WES: Whole Exome Sequencing
